# Knowledge mapping of COVID-19 and dentistry: A bibliometric analysis

**DOI:** 10.3389/fpubh.2022.1040175

**Published:** 2023-01-09

**Authors:** Jia Chen, Qian Zhang, Xin Liu, Ying Han, Qiming Gong

**Affiliations:** ^1^Xiangya School of Nursing, Central South University, Changsha, Hunan, China; ^2^Department of Neurosurgery, Xiangya Hospital, Central South University, Changsha, Hunan, China; ^3^National Clinical Research Center for Geriatric Disorders, Central South University, Changsha, Hunan, China; ^4^Department of General Practice, The Chinese People's Liberation Army 921 Hospital of Joint Logistics Support Force, Changsha, Hunan, China; ^5^Department of Oral and Maxillofacial Surgery, Center of Stomatology, Xiangya Hospital, Central South University, Changsha, Hunan, China; ^6^Center for Medical Genetics and Hunan Key Laboratory of Medical Genetics, School of Life Sciences, Central South University, Changsha, Hunan, China; ^7^Department of Nephrology, The Affiliated Hospital of Youjiang Medical University for Nationalities, Baise, China

**Keywords:** COVID-19, dentistry, oral surgery medicine, bibliometric analysis, CiteSpace, VOSviewer

## Abstract

**Background:**

COVID-19 has a significant impact on dental medicine. The present study aims to overview dental-related research on COVID-19 by visual mapping method.

**Methods:**

We analyzed the publications in the “Dentistry Oral Surgery Medicine” category in the Web of Science core collection. On June 10, 2022, we conducted an advanced search using the items TS = (“Novel coronavirus 2019” or “COVID 19” or “Coronavirus disease 2019” or “2019-nCOV” or “SARS-CoV-2” or “coronavirus-2”) and WC = (“Dentistry Oral Surgery medicine”) to screen publications in the dental field that focus on COVID-19 or SARS-CoV-2. The contributions of authors, journals, institutions, and countries were described using Microsoft Excel 2010 and VOSviewer. The keywords co-occurring analysis and references analysis were visualized using VOSviewer and CiteSpace.

**Results:**

A total of 1,732 papers were identified between 2020 and 2022. The United States, the United Kingdom, and Brazil were three major contributors to this field. Univ São Paulo (Brazil) ranked first with 55 publications in this field. Martelli Junior, Hercilio from Universidade Jose do Rosario Vellano (Brazil) was the most prolific author with 19 publications. *Oral Diseases* and *British Dental Journal* were the two most productive journals. The central topics were dental practice and infection control, oral manifestation related to COVID-19, dental education and online learning, teledentistry, and mental health problems.

**Conclusion:**

The growth rate of publications regarding dental research on COVID-19 has risen sharply. Research topics shifted from “Dental practice and infection control, oral manifestation related to COVID-19” in 2020 to “Dental education and online learning, teledentistry, mental health problems,” which are three important research topics for the future.

## 1. Introduction

Coronavirus disease 2019 (COVID-19) is currently sweeping the globe, and new cases are being reported daily (1). As of 17 June 2022, WHO has reported 535,863,950 confirmed cases of COVID-19, including 6,314,972 deaths [https://covid19.who.int/]. Academic scholars in the dental community actively respond to this unprecedented crisis caused by COVID-19. These researchers made a considerable effort into the transmission process, clinicopathological issues, diagnostic tools, prevention policy planning, and education reform approaches. For example, Costa et al. (2) reported that poor oral health conditions were strongly associated with COVID-19 symptoms, higher risk for admission to the intensive care unit (ICU), and a higher mortality rate. Xu et al. (3) found that the human angiotensin-converting enzyme 2 (ACE2) receptor has a higher expression level on the oral cavity mucosa and explained that the oral cavity is a potentially high-risk route for COVID-19 infection. COVID-19 is spread through direct contact with people and through saliva, blood, other body fluids, and contaminated surfaces that may carry the SARS-CoV-2 virus. Several scholars discussed how dental practices are at a high risk of spreading COVID-19 and outlined their experience in infection control in dental practices (4–6). Furthermore, dental education faced new challenges during the COVID-19 pandemic and lockdown. Iyer et al. (7) were concerned about the problems in dental teaching and learning during the COVID-19 pandemic and suggested that distance education (online learning) is an alternative solution for face-to-face dental education. Altogether, several aspects of COVID-19, and related papers have been published in dental discipline in the last 2 years. However, reading a few articles is insufficient to provide readers with an overview and key information. Specifically, new-researchers may be confused by the big data in this exploding and ongoing field. Thus, it is important to quickly find the significant publications, grasp the state-of-the-art, and new trend in this field.

Bibliometric analysis and text mining are widely accepted for analyzing big data in a particular field (8). Typically, bibliometric tools (e.g., VOSviewer, CiteSpace, Bibliometrix R, and Gephi, Pajek) are applied to analyze and visualize publications trends, prolific contributors, central themes, and research frontiers in a particular field (9). In 2020, two publications provide readers with an overview of dentistry and oral research on COVID-19by scientific mapping (10, 11). However, the included data in these two publications are relatively small (only 659 and 296). Moreover, they did not analyze this field's evolution and potential research frontiers. Because of the hundreds of papers published in 2021 and 2022 and the pandemic is still ongoing, the results of these two bibliometric analyses should be updated and future research direction should be pointed out. Thus, in this study, we utilized Citespace and VOSviewer, the most popular bibliometric tools, to visualize the state-of-the-art dentistry and oral research on COVID-19. We aim to reveal the following research questions (RQ).

RQ 1. The trend in publishing dentistry and oral research on COVID-19.
RQ 2. The most influential articles and primary contributors (e.g., authors, institutions, countries, and journals) for dentistry and oral research on COVID-19.
RQ 3. The hot themes and research frontiers for dentistry and oral research on COVID-19.

## 2. Materials and methods

### 2.1. Search strategy

Due to many diseases in the dentistry discipline, it may be difficult to include all dental diseases by using the search terms “oral or dental”. In addition, we chose the Web of Science Core Collection as our data resource to meet the data format standard in both CiteSpace and VOSviewer. Thus, we analyzed the publications in the “Dentistry Oral Surgery Medicine” category in the Web of Science core collection (WoSCC). On June 10, 2022, we conducted an advanced search using the items TS = (“Novel coronavirus 2019” or “COVID 19” or “Coronavirus disease 2019” or “2019-nCOV” or “SARS-CoV-2” or “coronavirus-2”) and WC = (“Dentistry oral surgery medicine”) to screen publications in the dental field that focus on COVID-19 or SARS-CoV-2. In order to eliminate irrelevant papers, two researchers (JC and QZ) independently searched the database and screened the title and abstract. For example, documents primarily centered on SARS or MERSE were removed. Finally, the senior dentists (YH, GM, and XL) reviewed the search results and discussed with the former individuals to create a final data version.

### 2.2. Data extraction and bibliometric analysis

The bibliometric parameters (e.g., title, keywords, journal, publication year, citations, author, institution, country, and references) were extracted. The data was then imported into Microsoft Excel 2010 (Redmond, Washington, USA) and VOSviewer (Leiden University, Leiden, the Netherlands) to determine who contributed the most (e.g., prolific authors, and institutions, countries). In VOSviewer, node size is positively related to the number of articles. Co-authorship analysis evaluates the relationship of collaboration between different countries, authors, and institutions (12). Cooperation strength positively correlates with the total link strength (TLS) between two nodes. The co-occurring and reference analysis keywords were visualized using CiteSpace (Version 5.8. R1) and VOSviewer (13).

## 3. Results

### 3.1. General data

**Figure 1:**
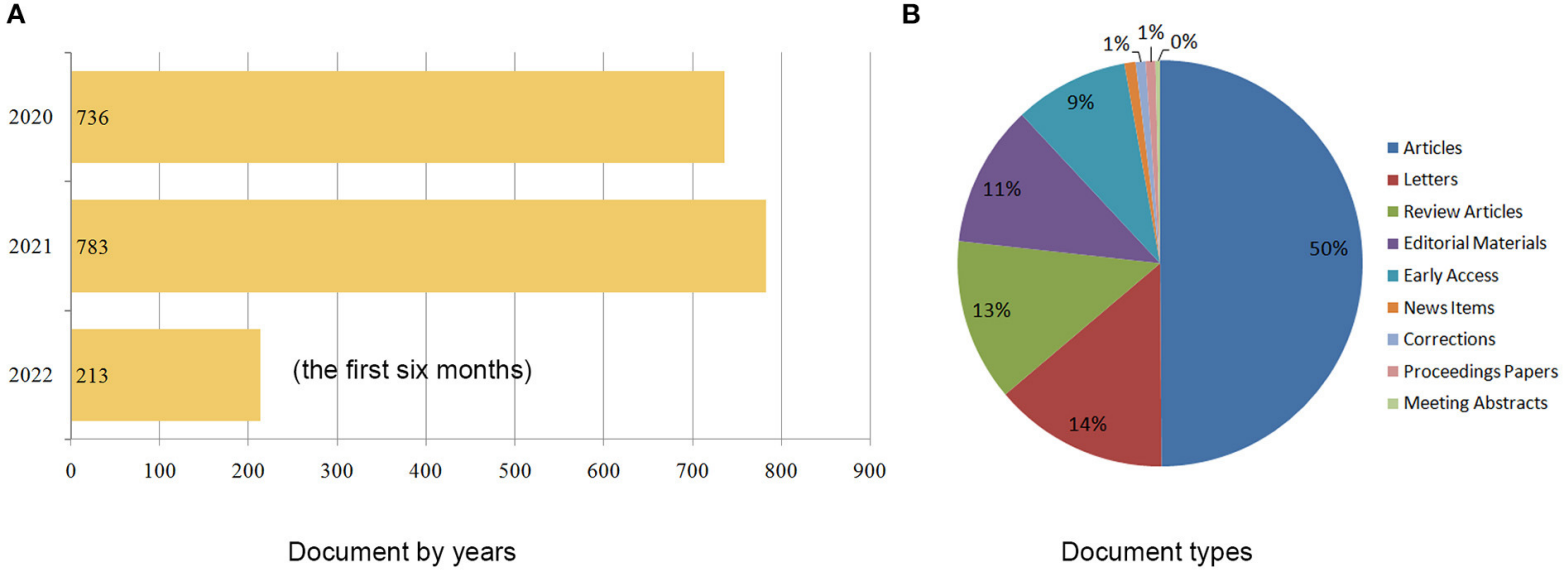
Distribution of publications by year **(A)** and type **(B)**.

A total of 1,732 papers were included in the final analysis. Figure 1A reveals the trend in the number of publications. The number of articles published in 2020 and 2021 are close, with more than 700. However, only 213 papers were published in the first half of 2022. Figure 1B shows the distribution of document types. Fifty percent were original articles, 14% were letters, and 13% were review articles. The total citations (TC) was 14,361, citations per paper (CPP) was 8.29, and H-index was 48. Altogether, 97 countries/regions, 1,844 institutions, 6,335 authors, and 137 journals contributed to producing these publications.

### 3.2. Countries

**Figure 2:**
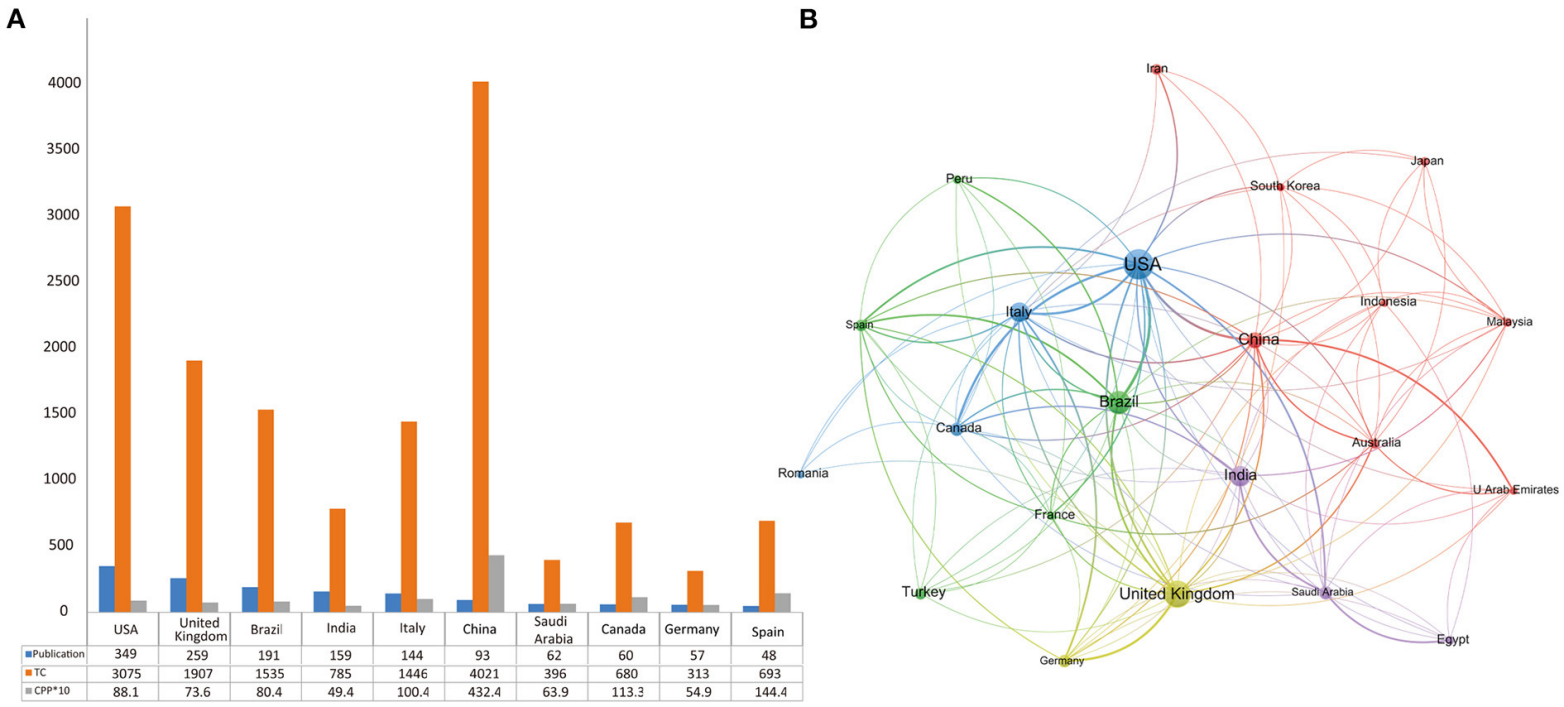
**(A)** Top 10 prolific countries, the number of publications, total citations (TC), and citations per publication (CPP) for each country. **(B)** Cooperation map of countries/regions. Node size indicates the number of articles. The width of links indicates the co-operation strength.

The top ten contributing countries and the network of most prolific countries are displayed in Figure 2. The USA topped the list with 349 publications (20.2% of total) and 3,075 TC. The UK (259 publications, 1,907 TC) and Brazil (191 publications, 1,535 TC) ranked second and third, respectively (Figure 2A). As for CPP, China, the USA, the UK ranked the top three. Figure 2B displays the network of most prolific countries. The minimum number of publications was 20. The USA, the UK, Brazil, India, Italy, and China were highlighted in the map, indicating their close collaborations and tremendous academic influence in this field. On the country collaboration map, five clusters were colored differently. The closer two countries are, the stronger their ties will be. The USA authors collaborated the most (20 countries), followed by the the UK authors (19 countries), and Italian authors (16 countries).

### 3.3. Institutions

**Figure 3:**
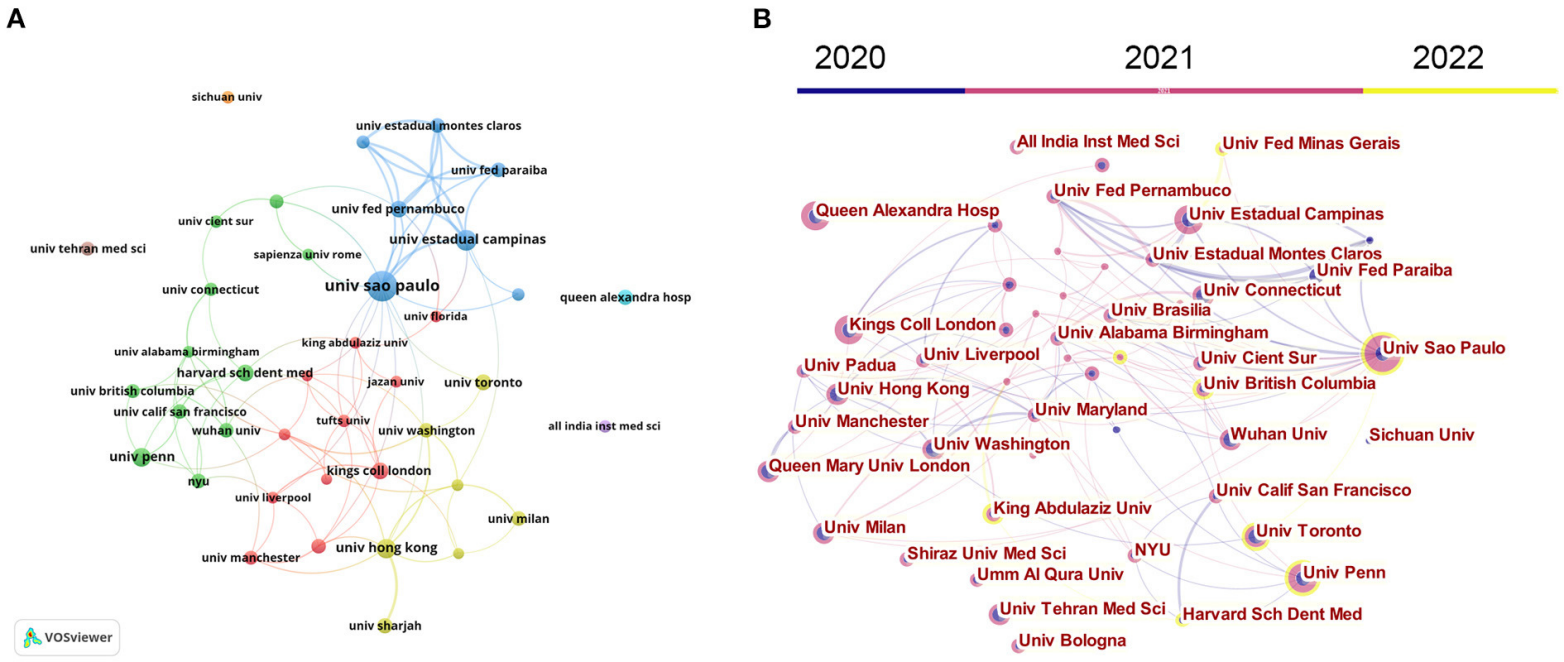
Co-operation map of the most productive institutions based on VOSviewer **(A)** and CiteSpace **(B)**. Node size indicates the number of publications. The link size refers to the co-operation intensity.

Figure 3 presents the collaboration of the most prolific institutions. Detailedly, Univ São Paulo (Brazil) ranked first with 55 publications. Univ Estadual Campinas (Brazil) ranked second with 28 publications, followed by Univ Hong Kong (China) with 25 publications. Regarding the citations, Sichuan Univ (China, 2,485 TC), Wuhan Univ (China, 828TC), and Univ São Paulo (Brazil, 527 TC) ranked in the top three. VOSviewer and CiteSpace visualized the institution's cooperation network. As demonstrated in Figure 3A, 40 institutions with at least ten publications were identified by VOSviewer, and Figure 3B also detected the active period of these institutions. Univ Washington, Univ Tehran Med Sci, Univ Estadual Campinas, Institutions in China (e.g., Sichuan Univ, Wuhan Univ, and Univ HongKong), and European (e.g., Kings Coll London, Queen Alexandra Hosp, Queen Mary Univ London, and Univ Milan) were more active from 2020 to 2021. On the other hand, Univ São Paulo, Univ Penn, Harvard Sch Dent Med, King Abdulaziz Univ, and Univ British Columbia were more active from 2021 to 2022. Inter-institutional cooperation had a regional characteristic and was divided into three major clusters, North America (centered by Univ Penn and Univ Toronto), European (centered by Kings Coll London and Queen Alexandra Hosp), and South America (centered by Univ São Paulo and Univ Estadual Campinas).

### 3.4. Authors

**Figure 4:**
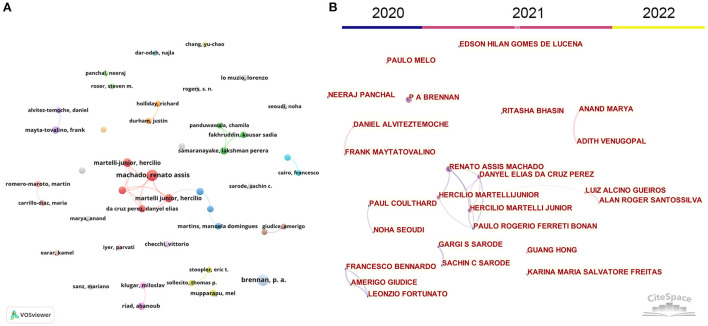
Co-operation map of the most productive authors based on VOSviewer **(A)** and CiteSpace **(B)**. Node size indicates the number of publications. The width of links indicates the co-operation strength.

Table 1 lists the top ten authors, and Figure 4 illustrates the collaboration of the most prolific authors. The most prolific author was Martelli Junior, Hercilio from Universidade Jose do Rosario Vellano (Brazil), with 19 publications. As for the most influential authors, Siqueira, WL (6 publications, 250 TC, 41.6 CPP) from the University of Saskatchewan (Canada) ranked first. The author's cooperation is visualized in Figure 4. As revealed in Figure 4A, 43 authors with at least five papers were recognized by VOSviewer. Figure 4B presents the active period of these authors. Only sporadic collaborations exist around several prolific authors (e.g., Martelli Junior, Hercilio, Machado, Renato Assis, Samaranayake, Lakshman Perera, and Mupparapu, Mel). Furthermore, these authors were active in 2020 and 2021 but not in 2022.

**Table 1:** Top 10 most prolific authors.

**Rank**	**Author**	**Publication**	**TC**	**CPP**	**Institution**	**Country**
1	Martelli Junior, Hercilio	19	127	6.7	Universidade Jose do Rosario Vellano	Brazil
2	Brennan, Peter A.	16	146	9.1	Queen Alexandra Hospital	UK
3	Machado, Renato Assis	16	116	7.3	Univ São Paulo	Brazil
4	Ferreti Bonan, Paulo Rogerio	8	109	13.6	Universidade Federal da Paraiba	Brazil
5	Riad, Abanoub	8	115	14.4	Masaryk University Brno	Czech Republic
6	Samaranayake, Lakshman Perera	8	162	20.3	University of Hong Kong	China
7	Da Cruz Perez, Danyel Elias	7	68	9.7	Universidade Federal de Pernambuco	Brazil
8	Gueiros, Luiz Alcino	7	80	11.4	Universidade Federal de Pernambuco	Brazil
9	Mayta-Tovalino, Frank	7	3	0.4	Universidad Cientifica del Sur	Peru
10	Mupparapu, Mel	7	18	2.57	University of Pennsylvania	USA

TC, total citations; CPP, citations per publication.

### 3.5. Journals

Table 2 lists the top ten active and co-cited journals. The top three most prolific journals were *Oral Diseases* (145 publications, 1,208 TC, 8.3 CPP), *British Dental Journal* (119 publications, 636 TC, 5.3 CPP), and *Journal of Dental Education* (105 publications, 762 TC, 7.3 CPP). More articles, however, do not imply a strong scientific influence. Thus, we also identified the top ten co-cited journals. *Journal of Dental Research, Lancet*, and *New England Journal of Medicine* were listed as the top three journals. Notably, five journals appeared in the list of prolific journals and co-cited journals (e.g., *Oral Diseases, Journal of Dental Research, British Dental Journal, Journal of Dental Education, and Journal of The American Dental Association*).

**Table 2:** The top 10 prolific journals and co-cited journals.

**Rank**	**Journal**	**Publication**	**TC**	**CPP**	**IF (2020)**	**JCR**	**Co-citation journal**	**Co-citations**
1	Oral Diseases	145	1208	8.3	3.511	Q1	Journal of Dental Research	895
2	British Dental Journal	119	636	5.3	1.6	Q4	Lancet	882
3	Journal of Dental Education	105	762	7.3	2.264	Q3	New England Journal of Medicine	857
4	Oral Oncology	81	730	9.0	5.337	Q1	British Dental Journal	733
5	British Journal of Oral & Maxillofacial Surgery	78	525	6.7	1.651	Q4	Journal of Dental Education	703
6	BMC Oral Health	54	258	4.8	2.757	Q2	Journal of The American Dental Association	684
7	Journal of The American Dental Association	52	262	5.0	3.634	Q1	Oral Diseases	602
8	Journal of Dental Research	50	1592	31.8	6.116	Q1	International Journal of Environmental Research and Public Health	561
9	Journal of Oral And Maxillofacial Surgery	36	230	6.4	1.895	Q3	JAMA-Journal of the American Medical Association	525
10	European Journal of Dental Education	35	303	8.7	2.355	Q2	International Journal of Oral Science	469

TC, total citations; CPP, citations per publication; IF (2020), impact factor; JCR, journal citation rank.

### 3.6. Top cited publications

Table 3 lists the top ten most cited publications. Four are original articles, four are reviews, and two are letters. Xu et al. produced the highest citation paper published in *International Journal of Oral Science*, entitled “*High expression of ACE2 receptor of 2019-nCoV on the epithelial cells of oral mucosa*” with 1280 TC (3). Most publications discussed about the impact of COVID-19 on dental practice (4–6) and dental education (5, 7). Several publications focused on the invasive mechanism of SARS-CoV2 through the oral cavity (3) or salivary glands (14, 15).

**Table 3:** Top 10 cited publications.

**Rank**	**First author**	**Article title**	**Journal**	**Document type**	**TC**	**Publication year**
1	Xu, Hao	High expression of ACE2 receptor of 2019-nCoV on the epithelial cells of oral mucosa	International Journal of Oral Science	Article	1,280	2020
2	Peng, Xian	Transmission routes of 2019-nCoV and controls in dental practice	International Journal of Oral Science	Review	864	2020
3	Meng, L	Coronavirus Disease 2019 (COVID-19): Emerging and Future Challenges for Dental and Oral Medicine	Journal of Dental Research	Article	713	2020
4	Ather, Amber	Coronavirus Disease 19 (COVID-19): Implications for Clinical Dental Care	Journal of Endodontics	Review	288	2020
5	Sabino-Silva, Robinson	Coronavirus COVID-19 impacts to dentistry and potential salivary diagnosis	Clinical Oral Investigations	Letter	219	2020
6	Iyer, Parvati	Impact of COVID-19 on dental education in the United States	Journal of Dental Education	Review	177	2020
7	Guo, Huaqiu	The impact of the COVID-19 epidemic on the utilization of emergency dental services	Journal of Dental Sciences	Article	175	2020
8	Izzetti, R	COVID-19 Transmission in Dental Practice: Brief Review of Preventive Measures in Italy	Journal of Dental Research	Review	173	2020
9	Coulthard, Paul	Dentistry and coronavirus (COVID-19)-moral decision-making	British Dental Journal	Article	161	2020
10	Xu, J	Salivary Glands: Potential Reservoirs for COVID-19 Asymptomatic Infection	Journal of Dental Research	Letter	137	2020

TC, total citations.

### 3.7. Co-occurrence of keywords

**Figure 5:**
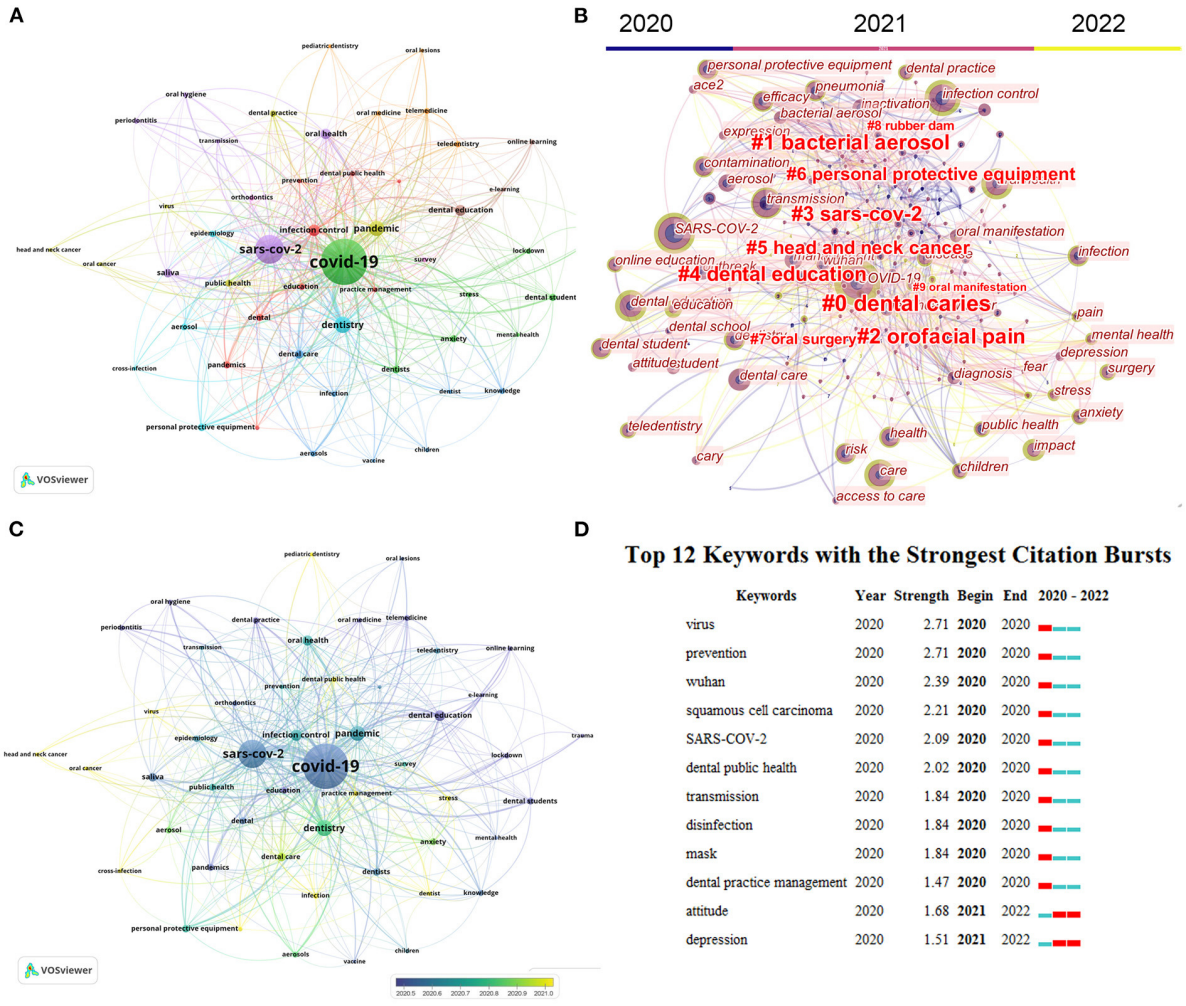
Analysis of author keywords. **(A)** The co-occurrence networks of keywords are visualized by VOSviewer. Large nodes represented keywords with high frequency; the same color indicates closer relationships; **(B)** Chronological overview of the co-occurrence network of author keywords. Dark blue refers to an earlier appearance, and yellow refers to the latest appearance. **(C)** Keywords clusters named by CiteSpace LLR algorithm from 2020 to 2022. **(D)** Top 10 keywords with the strongest citation bursts. The red bar indicates the burst duration. The burst strength refers to the importance of the keyword to the research field.

Using CiteSpace and VOSviewer, we analyzed the co-occurrence of keywords and key references in this field. To remove the duplicate keywords with a similar meaning, we merged these keywords using the file “Thesaurus (VOSviewer) and CiteSpace.Alias (CiteSpacce)” (Supplementary Table 1). For instance, COVID 19 was replaced by COVID-19, coronavirus-2 was replaced by SARS-CoV-2. Figure 5A presents the keywords co-occurrence map (VOSviewer). The top ten keywords with the highest occurrences are “COVID-19 (789 times), SARS-CoV-2 (339 times), dentistry (123 times), pandemic (114 times), infection control (68 times), dental education (61 times), oral health (55 times), and saliva (42 times), personal protective equipment (32 times), and anxiety (29 times).” Furthermore, Figure 5B demonstrates keyword co-occurrence and keywords clusters identified by CiteSpace. They are “dental caries, bacterial aerosol, orofacial pain, dental education, head, and neck cancer, personal protective equipment, oral surgery, rubber dam, and oral manifestation.” We used the keywords overlay map in VOSviewer (Figure 5C) and CiteSpace's keywords burst (Figure 5D) to detect the evolution of hot topics. In 2020, the major topics were centered on the management of dental practice, dental education, and potential transmission route through the oral cavity. In 2021 and 2022, the hot topics shifted to dentists' and dental students' attitudes toward public health and mental health (e.g., anxiety, depression, stress).

### 3.8. Co-citation references

**Figure 6:**
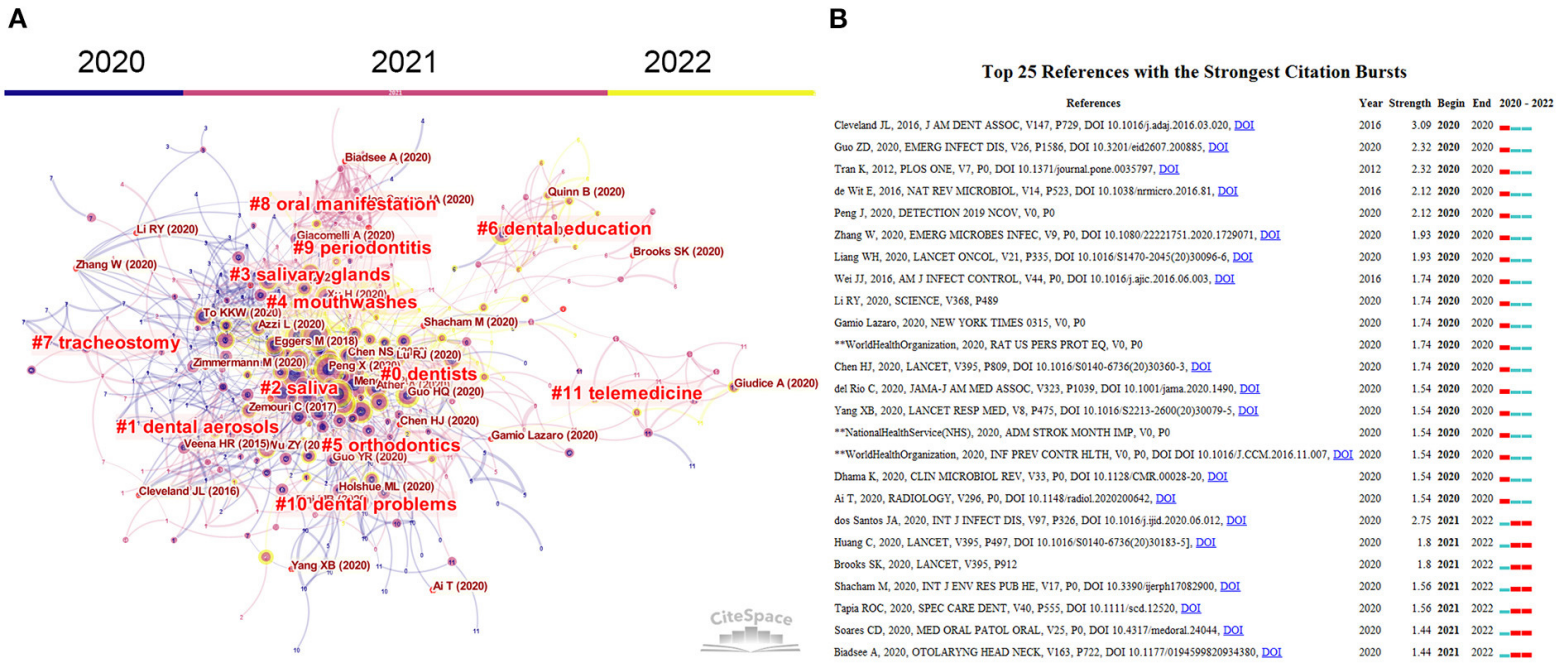
**(A)** Reference co-citation network clustered by CiteSpace. The nodes and links are distinguished by colors, in which dark color refers to an earlier co-citation relationship. References with at least 100 citations are displayed in the network in nodes named by the first author (publication year). The size of the node positively relates to the citation number. The red writing reports the name of the cluster that is auto-identified by the Citespace LLR algorithm. **(B)** Top 25 references with the strongest bursts. The red bar indicates the burst duration. The burst strength suggests the importance of this article to the research field.

Scientific development is based on previous research. We used CiteSpace's co-citation references and reference bursts to find key references in this field. As displayed in Figure 6A, 11 clusters focus on various topics. Such as “dentists practice, dental aerosols, saliva glands, mouthwashes, orthodontics and periodontitis, dental education, oral manifestation, telemedicine”. Key references were presented as the type of “author name (year),” Like “Cleveland et al. (16)” and “Biadsee et al. (17)”. Additionally, to assess the evolution of knowledge network in this field, we used the burst module in CiteSpace to identify crucial references. Figure 6B indicates the top 25 references with the highest citation burst. Cleveland et al. published the document with the highest citation burst (*n* = 3.09). In this paper, the authors only discovered three articles that reported the transmission of bloodborne pathogens (hepatitis B and C viruses) during dental practice before 2015. They concluded that the risks of transmission during dental practice are due to handpieces were not heat sterilized between patients, volunteers were not trained on bloodborne pathogens, and dentists used unsafe injection methods (16). Seven publications (citation bursts ending in 2022) attracted increasing attention in 2022. They are “Amorim dos Santos et al. (18), Godeau et al. (19), Brooks et al. (20), Shacham et al. (21), Cruz Tapia et al. (22), Soares et al. (23), Biadsee et al. (17).”

## 4. Discussion

The COVID-19 outbreak has triggered an unprecedented global healthcare crisis, including dentistry and oral discipline (24). As a result, many papers were produced to characterize the impact of COVID-19 on the dentistry and oral discipline. In this study we examined the hot topics and research trends in this field using bibliometric analysis. The growth rate of publications has risen sharply. The number of papers produced in 2021 was nearly threefold that in 2020. By the first 6 months of 2022, 1079 articles were also published, a moderate decline trend. *In vivo* and *in vitro* studies show that with the new variants emerging (Delta and Omicron), the pathogenicity of SARS-CoV-2 was milder compared with the ancestral strain (25, 26). Several countries abandoned strict health policies aimed at preventing and controlling COVID-19. For example, British Prime Minister Boris Johnson announced that from February 24, 2022, all COVID-19 prevention and control measures in Britain would be suspended, and launched the “Living with COVID-19” program. CDC in the USA also changed their COVID-19 guideline from “preventing infections” to “prevent severe infected” and especially “protecting vulnerable groups for severe illness” (https://www.cdc.gov/mmwr/volumes/71/wr/mm7133e1.htm?s_cid=mm7133e1_w). Nonetheless, the COVID-19 pandemic may have a lasting impact on society, science, and education. As a result, we anticipate that more publications describing the impact of COVID-19 on dentistry will be published in the future.

### 4.1. Performance analysis

A performance analysis is quite common among literature review studies. It is invaluable to emerging scholars because it informs them about where to look for quality research and how to select research partnerships. Additionally, veterans consider the performance analysis positively because it gives them an opportunity to gain recognition for their contributions in terms of productivity and influence and steering their research directions. This study used citations and publications as measures of influence and productivity and showed the geographical distribution and co-authorship analysis.

#### 4.1.1. Geographical distribution

The publications from different countries suggested that oral problems associated with COVID-19 are gaining global attention. In terms of citations, the USA and China got the most citations, which indicated scholars in this field have widely recognized their work on oral problems. The USA, the UK, and Brazil contributed the most publications in this field, which may reflect the growing number of infected people in these countries. These countries were more concerned about oral health than other countries. Therefore, the future step may focus on oral problems in African and Middle Asian countries during the COVID-19 pandemic. We found that the University of São Paulo has published far more articles in this field (*n* = 55) than any other institution. Scholars at this institution studied multiple aspects of COVID-19 in dentistry and oral discipline. For example, dos Santos et al. investigated oral manifestations in patients with COVID-19 (12). Brandaoet al. discussed whether oral keratinocytes and minor salivary glands could be the potential targets of SARS-CoV-2 (27); Providing dental care for children during COVID-19 (28); Social media and telemedicine for oral diagnosis (29); COVID-19 pandemic and the impact on dental education was also explored (30, 31).

#### 4.1.2. Co-authorship analysis

Understanding the dynamics of authors is critical to learning how new scholars conduct their future research. Authors' collaborations also stand behind inter-institutional and international cooperation. We used VOSviewer and CiteSpace to present the cooperation between prolific countries, institutions, and authors. However, in this study, only several research groups performed relatively active cooperation with other groups.

Author group #1 Machado, RA and Martelli JH et al. This is the largest author group with the most collaboration. Machado, Renato Assis, and Martelli Junior, Hercilio are central to this author group. These authors responded quickly to COVID-19 and published a series of articles to discuss the potential impact of COVID-19 on oral health (32), dental education (30, 33), and telemedicine for oral diagnosis and counseling (29).

Author group #2 Samaranayake et al. This is the second largest author group with the most collaboration. Samaranayake LP is the most impactful author. Most of their publications are reviews. As a result, fewer author collaborations exist than in the previous largest author group. They focused on COVID-19 transmission control in dentist clinic practicing (34–36), and prodromal symptoms of dental on COVID-19 (37, 38).

### 4.2. Emergent research frontiers

Co-citation means that two works are cited together, usually a sign of knowledge similarity (39). The VOSviewer keyword co-occurrence overlay map and CiteSpace citation burst are frequently used to visualize knowledge evolution and potential research frontiers (40, 41). In the following discussion, we extend our suggestions for future research based on these clusters.

**Cluster#1** dental practice and infection control: Due to the specificities of dental interventions, such as aerosol generation, sharps handling, and the proximity of providers to the patient's oropharynx, dental providers and patients in dental offices are at high risk for SARS-CoV-2 infection. Meng et al. (5), Ather et al. (6) and Peng et al. (4) overviewed the epidemiology, symptoms, and transmission routes of SARS-CoV-2 and provided specific dental treatment recommendations. First, telescreen patients with oral problems and triage them urgently. Elective dental care should be postponed for at least 2 weeks for potential SARS-CoV-2-positive patients. Second, dental professionals should use non-contact thermometers to measure the body temperature of patients when they arrive at the dentist's office, and patients should complete a detailed medical history form regarding COVID-19 screening. Finally, dentists should follow the following recommendations during clinic practice.: 1. appropriate use of personal protective equipment and hand hygiene practices; 2. preprocedural mouth rinse with 0.2% povidone-iodine or 0.5–1% hydrogen peroxide; 3. use disposable devices as much as possible; 4. use a rubber dam to minimize splatter generation; 5. operate in airborne infection isolation rooms/negative-pressure treatment rooms; 6. reduce the use of ultrasonic instruments, high-speed handpieces; 7. prevent perforation and cross contamination during intraoral and extraoral imaging using double barriers; 8. disinfect the dental clinics using chemicals regularly and manage the medical waste.

**Cluster#2** oral manifestation related to COVID-19: Patients with COVID-19 often hyposmia and dysgeusia (42, 43). Moreover, several papers reported dry mouth and oral mucosal changes (e.g., petechiae, ulcers, plaque-like lesions, reactivation of herpes simplex virus 1, geographical tongue, and desquamative gingivitis) (44). Hence, dental health professionals should be aware of these oral manifestations to better evaluate and screen potential COVID-19-positive patients. Farid et al. (44) and Amorim et al. (45) summarized and updated the oral manifestations of COVID-19. The prominent locations for mucosal lesions are tongue, palate, and labial mucosa because angiotensin-converting enzyme 2 (ACE2) cell receptors are abundant on the oral mucosa and may be the target of SARS-CoV-2 invasion (15). The prevalence of taste disorders in patients with COVID-19 was 45–60% (12, 44, 46). However, the association between taste disorders and the severity of COVID-19 and female COVID-19 patients had low confidence, indicating that more research is needed to validate the causality. Additionally, the prevalence of oral adverse effects was slightly higher in the COVID-19 vaccine group than in the seasonal influenza group, but no significant statistical difference was observed (47).

**Cluster#3** dental education: During the COVID-19 pandemic, dental schools faced a significant challenge in protecting the health of students, faculty, and patients while keeping in line with epidemic prevention policies and ensuring student education continuity (7). During the initial outbreak of COVID-19, Wuhan conducted a lockdown policy to curb the spread of SARS-CoV-2. Thus dentist students could not undergo face-to-face clinical training. Meng et al. (5) proposed that teachers conduct online lectures and encourage students to fully utilize online resources. Furthermore, teachers should focus on the pressure associated with COVID-19 and provide students with psychological services. Di Carvalho Melo et al. (48) comprehensively reviewed the frequency of different teaching methodologies, tools, and platforms applied in dental education during the COVID-19 pandemic. The advantages of online learning are the comfort of attending classes everywhere and every time, the possibility of less distraction, greater concentration on presentations, and the opportunity to review recorded classes repeatedly to take additional notes. However, there are disadvantages as well, such as exhaustion from excessive screen time, lack of human interaction, and distractions from home life. Technical difficulties have also been reported, such as unstable internet connection and video and audio issues. Stress and anxiety were also reported, the doubt about graduation completion, and the fear of infection. Amir et al. (49) reported that only 44.2% of students preferred distance learning over classroom learning, although these students agreed that distance learning was a more efficient learning method (52.6%). Besides students' problems, teaching staff also faced challenges, such as inexperienced dealing with technology, internet connection, and content transition to online education (50). Online lectures or demonstrations have become an unavoidable method of dental education in the future (51). However, as dental education is an applied discipline, it cannot be taught only *via* online learning. Thus, virtual simulation teaching using virtual reality systems and haptic technology can be investigated in the future.

**Cluster#4** teledentistry: Reducing physical contact is necessary to reduce the risk of infection, therefore telemedicine is an alternative for dental health workers and patients to ensure the continuity of oral care (52). Several studies applied teledentistry in coping with the COVID-19. Smith et al. (53), evaluated the symptoms of COVID-19 patients and triage these patients for better continuity of dental care by telemedicine. Additionally, teledentistry could be used to monitor and follow up on patients. Alsafwani et al. (54) reported that 65% of patients got a median improvement in oral pain after being consulted *via* teledentistry during the COVID-19 pandemic. Morishita et al. (55) reported that teledentistry could help patients get dental services and dental care for the first visit and a follow-up visit in Japan. The best advantage of teledentistry is that it makes patients get dental care services quickly and enables the exchange of information between professionals, which is helpful in the precision of diagnosis and therapy. However, it also has several limitations, such as the inability to perform physical examinations and conduct complementary examinations remotely.

Furthermore, effective teledentistry necessitates a solid infrastructure on both ends to ensure video quality and images. Kilipiris et al. investigated parental satisfaction with telemedicine in the follow-up of children during the COVID-19 pandemic. The authors reported that 72.3% of parents were satisfied with the telemedicine program, and 67.2% found it convenient. However, only 18.7% of them would prefer telemedicine consultations in the future (56). As a result, it is critical to train clinicians in teledentistry and improve technical limitations to provide better tele-dental care in the future.

**Cluster#5** mental health problems. Stress is a common problem for dentists (57). During the COVID-19 pandemic, like other front-line healthcare workers, dental professionals can be affected by disease-associated fear, stress, and pressure (58–60). Estrich et al. (59) reported that 8.6% of dentists experienced depression, and 19.5% of dentists experienced anxiety after 6 months of the COVID-19 pandemic outburst in the USA. Collin et al. (58) compared the levels of psychological distress in the UK dentists before and during the COVID-19 pandemic. Interestingly, while 57.8% of dentists had psychological distress (over the clinical threshold), a 10% decrease was noticed compared to the results before the COVID-19 pandemic (61). Maybe the UK's lockdown and social isolation policy provide the time away from clinical practice. They could spend more time with family, indulge in hobbies, relax, and recharge. Contrary to this, mental problems in the UK have changed over time. Owen et al. (62) reported that 82% of dentists' increased stress levels in Wales. Working conditions and financial pressures caused by the pandemic have directly impacted the mental health of many dentists. Apart from dentist practitioners, dental students are also affected by the COVID-19 pandemic. Hakami et al. (60) reported that 60.64, 37.02, and 34.92% of students experienced elevated depression, anxiety, and stress at the beginning of COVID-19 lockdown. These problems highlight the importance of psychological support during major epidemic outbreaks and need reform to support dentist practitioners more time to themselves.

## 5. Limitation

Since the article search was conducted in the WoSCC, an article's inclusion or exclusion is subject to two main conditions. First, the article must have either “COVID-19” or “SARS-CoV-2” in the “title”, “abstract”, “author keywords”, and “KeywordPlus” (i.e., keywords assigned by the Web of Science). Second, the article must be published in journals focusing on Dentistry and Oral Surgery medicine. Undoubtedly, it will exclude several papers published in general medicine journals (e.g., *Lancet* and *JAMA*). Bibliometric studies typically rely on bibliographic data from the scientific database. Thus, any errors in the database could affect the dataset for the analysis. Despite the possibility of errors, the impact of such errors is likely negligible because the authors carefully checked and corrected for recognizable errors (e.g., missing data—e.g., author name). Additionally, it may yield selection bias due to several publications written in Japanese, Russian, Portuguese were excluded. However, the number of publications addressed in this study is large enough to analyze the emerging knowledge network and research trend.

## 6. Conclusion

This study comprehensively analyzed publications on COVID-19 in dentistry and oral surgery medicine. The growth rate of publications on dental research related to COVID-19 has increased dramatically. The research topics shifted from “Dental practice and infection control, oral manifestations related to COVID-19” to “Dental education and online learning, telemedicine, and mental health issues” in 2021, three important future research topics.

## Data availability statement

The original contributions presented in the study are included in the article/Supplementary material, further inquiries can be directed to the corresponding authors.

## Author contributions

QZ and YH conceived of the study, participated in its design, and drafted the manuscript. JC involved in study design, obtained data and visualization, and helped to draft the manuscript. QG, YH, and XL provided the theoretical frameworks, supervised the process of this study, and critical edited and reviewed the manuscript. All authors read and approved the final manuscript.
