# Supplementary material for: Knowledge mapping of COVID-19 and dentistry: A bibliometric analysis
Source: Front Public Health. 2023 Jan 9;10:1040175. doi: 10.3389/fpubh.2022.1040175 (PMC9868823; doi:10.3389/fpubh.2022.1040175)
Supplement: Supplementary file 1 [file Table_1.docx]

Table S1. Thesaurus

| Label | Replaced by |
| --- | --- |
| coronavirus disease 2019 | covid-19 |
| coronavirus disease 2019 (covid-19) | covid-19 |
| covid | covid-19 |
| covid 19 | covid-19 |
| 2019-nCOV | covid-19 |
| covid-19 pandemic | covid-19 |
| coronavirus | sars-cov-2 |
| converting enzyme 2 | ace2 |
| coronavirus infections | covid-19 |
| covid-19 pandemic | covid-19 |
| sars | sars-cov-2 |
| sars-cov-2 infection | sars-cov-2 |
| coronavirus-2 | sars-cov-2 |
| sars-cov2 | sars-cov-2 |
| severe acute respiratory syndrome coronavirus 2 | sars-cov-2 |
| health care workers | health care worker |
